# Predictors of cognitive impairment in drug-resistant epilepsy: the role of interictal EEG abnormalities

**DOI:** 10.1186/s42494-026-00244-8

**Published:** 2026-02-04

**Authors:** Saleh Baeesa, Fawzi Babtain, Ahmad Albeshri, Amal Alkhotani, Rakan Bokhari, Motaz Fadul, Mohammed Karami, Mazen Basheikh, Adnan Badahdah, Ahmed Bamaga, Mohammed Alshurem, Raed Gasemaltayeb, Wareef Alzahrani, Ahmed Najjar, Yasser Alamri, Humaira Waseem, Amber Hassan, Maher Kurdi

**Affiliations:** 1 Department of Neurosciences, King Faisal Specialist Hospital and Research Center, Jeddah, 21499 Saudi Arabia; 2 Faculty of Medicine, Alfaisal University, Riyadh, Saudi Arabia; 3 Department of Neurosurgery, King Abdulaziz Medical City, National Guard Hospital, Jeddah, 21423 Saudi Arabia; 4 Department of Medicine, College of Medicine, Umm Al-Qura University, Makkah, 21955 Saudi Arabia; 5 Department of Surgery, Faculty of Medicine, King Abdulaziz University, Jeddah, 21589 Saudi Arabia; 6 Department of Pathology, Faculty of Medicine, King Abdulaziz University, Rabigh, 21911 Saudi Arabia; 7 Department of Clinical Physiology, Faculty of Medicine, King Abdulaziz University, Jeddah, 21589 Saudi Arabia; 8 Department of Internal Medicine, Faculty of Medicine, University of Jeddah, Jeddah, 23218 Saudi Arabia; 9 Department of Pediatrics, Faculty of Medicine, King Abdulaziz University, Jeddah, 21589 Saudi Arabia; 10 Department of Neurology, College of Medicine, Imam Abdulrahman Bin Faisal University, Dammam, 31441 Saudi Arabia; 11 Department of Internal Medicine, Faculty of Medicine, King Abdulaziz University, Rabigh, 21911 Saudi Arabia; 12 Department of General and Specialized Surgery, College of Medicine, Taibah University, Madinah, 42351 Saudi Arabia; 13 Department of Neurology, King Salman Bin Abdulaziz Medical City, Madinah, 42317 Saudi Arabia; 14 Department of Data Science, INTI International University, Nilai, 71800 Malaysia; 15 European School of Molecular Medicine, University of Milan, Milan, 20122 Italy; 16 Translational Neuroscience Lab, CEINGE Biotechnologies Avanzate S.c.a. r.l., Naples, 80145 Italy

**Keywords:** Drug-resistant epilepsy, Interictal electroencephalograph, Cognitive impairment, Montreal Cognitive Assessment, Predictors

## Abstract

**Background:**

Cognitive impairment (CI) affects approximately one-third of patients with drug-resistant epilepsy (DRE), underscoring the need for accessible predictors. Interictal electroencephalographic (EEG) abnormalities have been proposed as potential indicators of cognitive dysfunction; however, their independent diagnostic utility is unclear. This study aimed to investigate the association between interictal EEG patterns and CI in adults with DRE, with a specific focus on evaluating their incremental predictive value beyond established clinical predictors.

**Methods:**

In this cross-sectional study of 90 adults with DRE were recruited over a six-month period. Participants were stratified into two groups based on their Montreal Cognitive Assessment (MoCA): those with cognitive impairment (Cases; *n* = 45; MoCA < 26) and those with preserved cognition (Controls; *n* = 45; MoCA ≥ 26). All participants underwent routine interictal scalp EEG, An EEG recording was classified as abnormal if epileptiform discharges or significant background slowing was identified. The relationships between cognitive status and various clinical variables-including age, monthly seizure frequency and epilepsy type were analyzed using multivariable logistic regression, with expressed as odds ratios alongside their 95% confidence intervals.

**Results:**

The frequency of monthly seizures was significantly higher in the CI group compared to the control group (9.6 ± 2.8 vs. 5.4 ± 2.1 seizures/month, *P* < 0.001). Interictal EEG abnormalities were also more prevalant in CI group (77.8% vs. 57.8%; OR = 2.56, 95% CI: 1.02–6.41, *P* = 0.041). However, in the adjusted multivariable model, only seizure frequency reained a signifcant independent association with CI (adjusted OR = 0.46, 95% CI: 0.32–0.65, *P* < 0.001), indicating that EEG abnormalities did not confer significant additional predictive power after accounting for seizure burden.

**Conclusions:**

Seizure burden emerged as the predominant predictor of CI with DRE, while interictal EEG abnormalities demonstrated a univariate correlation with cogntive status, this association was not independent in the adjusted analysis. EEG findings may still provide contextual or supportive clinical context, emphasize that a comprehensive approach integrating seizure management with cognitive assessments is warranted, rather than relying primarily on interictal EEG for cognitive risk stratification.

## Background

Epilepsy is a chronic neurological disorder characterized by recurrent, unprovoked seizures arising from abnormal bursts of neuronal activity [1, 2]. It affects over 50 million individuals worldwide, imposing substantial health, psychological, and social burdens [3]. Seizures vary in their clinical presentation, ranging from subtle lapses in awareness to generalized tonic–clonic convulsions, and may originate from focal or generalized brain networks [3, 4]. Despite advanced in treatment, approximately one-third of patients with epilpsy develop drug-resistant epilepsy (DRE), defined by persistent seizures despite adequate trials of at least two appropriately selected and tolerated anti-seizure medications [5, 6]. Nevertheless, beyond the physical manifestations of seizures, epilepsy is frequently complicated by cognitive and psychosocial comorbidlities that further impair patients’ quality of life. Cognitive impairment (CI) represents one of the most prevalent and disabling of these comorbidities, affecting up to one-third of patients with epilepsy [7–9]. It substantially diminishes quality of life [10–12] and typically manifests as deficits in memory, attention, executive function, and learning [8, 13]. The development of CI is multifactorial, influenced by factors including seizure frequency, age at onset, disease duration, epilepsy type, history of status epilepticus, neurological deficits, and exposure to polytherapy [14–17]. Early-onset and frequent seizures are particularly detrimental as they may interfere with normal brain maturation and disrupt the acquisition of cognitive skills [15]. Consequently, routine cognitive assessment is increasingly recognized as an essential component of epilepsy management, aiding in diagnosis, ongoing monitoring, and rehabilitation planning [15, 16].

Electroencephalography (EEG) remains the cornerstone neurophysiological tool in epilepsy evaluation. Although abnormal interictal EEG patterns—such as epileptiform discharges and background slowing—have been associated with cognitive dysfunction in several studies, the evidence remains inconclusive [17, 18]. Some investigations highlight a direct association between interictal abnormalities and cognitive impairment, whereas others suggest that clinical factors such as seizure frequency, illness duration, and overall disease burden have a more substantial impact on cognitive decline [17–20]. Moreover, many prior studies have enrolled heterogeneous epilepsy populations, with limited specific focus on patients with DRE, who are at the highest risk for cognitive morbidity.

In this context, the present study was designed to investigate the association between interictal EEG abnormalities and CI in adults with DRE. By focusing on a homogeneous DRE cohort and employing a pragmatic, clinically applicable EEG classification alongside standardized cognitive screening, this study aims to evaluate whether interictal EEG findings independent value for cognitive risk stratification or primarily reflect the underlyingburden of seizures.

## Methods

A retrospective cross-sectional study was conducted, including 90 adults with DRE recruited from the outpatient neurology clinic from October 2024 to April 2025. This study was approved by the Ethical Review Committee [no. 183-Synopsis/Neurology-II/FJ/ERC], and all participants had provided written informed consent in accordance with the principles outlined in the Declaration of Helsinki. The inclusion criteria were age 18–65 years, with a confirmed diagnosis of epilepsy according to the International League against Epilepsy (ILAE) criteria, and fulfilment of DRE criteria that are defined as failure of at least two appropriately chosen and well-tolerated anti-seizure medications to achieve sustained seizure freedom [21]. Accordingly, all participants were receiving anti-seizure polytherapy at the time of EEG and cognitive assessment. However, the burden of anti-seizure medications was not included in the regression model due to the variability in drug combination and dosing, which was a limitation of this study. The exclusion criteria of participants included acute medical or surgical illness, significant systemic comorbidities unrelated to epilepsy, psychogenic seizures, substance abuse, and major psychiatric or neurodevelopmental disorders such as psychosis, dementia, or cerebral palsy with intellectual disability. The neurological deficits of DRE patients were defined as persistent focal neurological signs documented on clinical examination, including hemiparesis, aphasia, visual field deficits, or sensory impairments. Structural abnormalities were identified based on brain imaging reports, including hippocampal sclerosis, cortical dysplasia, gliosis, and encephalomalacia, corresponding to seizure localization.

The sample size was estimated using OpenEpi (Version 3, SSCC) for a 1:1 comparative design with EEG abnormality as the exposure variable, resulting in a total of 90 participants [22]. These participants were stratified into two groups based on cognitive status: (1) Cognitively impaired group (cases; *n* = 45), defined as Montreal Cognitive Assessment (MoCA) scores < 26, and (2) Cognitively preserved group (controls; *n* = 45), defined as MoCA scores ≥ 26. This binary stratification allowed clinically applicable comparisons of groups using a validated screening threshold, rather than to characterize domain-specific cognitive profiles.

### Cognitive assessment

Cognitive status was evaluated using MoCA, a 30-point screening tool assessing attention, executive function, memory, language, visuospatial ability, abstraction, calculation, and orientation [20]. MoCA scores < 26 indicate cognitive impairment, in line with previously validated studies. While MoCA does not permit detailed domain-level analysis, its use reflects routine clinical screening practice in epilepsy clinics and enables standardized categorization across heterogeneous patient populations.

### EEG assessment

During the six months of cognitive testing, all participants underwent a standardised interictal scalp EEG recording for 20–30 min using a digital EEG system (Nihon Kohden, Tokyo, Japan) with electrodes positioned according to the international 10–20 system. Electrode impedances were maintained below 5 mΩ, and standard bandpass filters of 1–70 Hz were applied. EEG signals were digitally acquired at a sampling frequency of 256 Hz and a notch filter at 50 Hz to eliminate power-line interference. No additional post-processing or automated filtering was applied beyond standard clinical settings. EEGs were reviewed independently by two experienced neurophysiologists who were blinded to the cognitive status. Artifact-free epochs of at least 10 s were visually inspected during analysis. Segmentation was performed only for interpretative purposes and not for automated quantitative analysis. EEGs were classified as normal or abnormal based on predefined clinical criteria. Abnormal EEG was indicated as the presence of epileptiform discharges (spikes, sharp waves, spike-and-wave complexes) and/or generalised or focal background slowing. This dichotomous EEG classification was intentionally adopted to mirror routine clinical reporting, acknowledging that it does not capture EEG burden, severity, or spectral complexity.

### Statistical analysis

Group differences were analyzed using chi-square/Fisher’s exact tests for categorical variables and *t*-tests for continuous variables. EEG findings were converted into a binary categorical variable, with normal EEGs coded as 0 and abnormal EEGs coded as 1. This coding enabled integration of EEG findings into logistic regression models with cognitive status measures. The logistic regression assessed associations between interictal EEG abnormalities and cognitive impairment, adjusting for key covariates. Odds ratios with 95% confidence intervals were reported, and significance was set at *P* < 0.05. All analyses were conducted using SPSS version XX (IBM Corp., Armonk, NY).

## Results

The mean age at epilepsy onset (40.3 ± 12.7 vs. 42.9 ± 14.7 years, *P* = 0.368) and the duration of illness (10.5 ± 3.4 vs. 9.2 ± 2.9 years, *P* = 0.060) did not differ significantly between the cognitively impaired group and the cognitively preserved group (Table 1). In contrast, the seizure frequency per month was significantly higher among the cognitively impaired patients (9.6 ± 2.8) compared with the cognitively preserved individuals (5.4 ± 2.1, *P* < 0.001). The MoCA scores were also markedly lower in the cognitively impaired group (20.8 ± 2.7 vs. 28.2 ± 1.3, *P* < 0.001).

**Table 1:** Demographics and clinical characteristics of the study participants

**Variable**	**Cognitively impaired group (***n* = 45**)**	Cognitively preserved group **(***n* = 45**)**	***P*-value**
Age at onset (years)	40.31 ± 12.67	42.93 ± 14.72	0.368
Duration of illness (years)	10.49 ± 3.40	9.22 ± 2.88	0.060
Seizure frequency per month	9.56 ± 2.78	5.36 ± 2.07	< 0.001
MoCA score	20.82 ± 2.71	28.18 ± 1.34	< 0.001

Mean ± SD

*MoCA* Montreal Cognitive assessment

The cognitively impaired patients experienced a more frequent history of status epilepticus than the cognitively preserved patients (51.1% vs. 31.1%), though the difference was not statistically significant (*P* = 0.087) (Table 2). Neurological deficits were also more common in the cognitively impaired group (62.2% vs. 51.1%, *P* = 0.395). With respect to the epilepsy type, temporal and frontal lobe epilepsies were almost similarly represented in the cognitively impaired group (44.4% vs. 46.7%), while frontal lobe epilepsy was the most predominant among cognitively preserved patients (71.1% vs. 26.7%). Occipital and parietal epilepsies had a low overall frequency, and the distribution of epilepsy types demonstrated a trend toward statistical significance (*P* = 0.099). Interestingly, abnormal interictal EEG findings were observed significantly more often among cognitively impaired patients compared with controls (77.8% vs. 57.8%; *P* = 0.041). The odds of having an abnormal EEG were 2.56 times higher in the impaired group (95% CI: 1.02–6.41) (Table 3). This association was observed at the unadjusted level, suggesting that EEG abnormalities may act as a supportive clinical marker rather than an independent predictor of cognitive impairment.

**Table 2:** Clinical characteristics of the study participants by group

**Variable**	**Category**	**Cognitively impaired group (***n* = 45**)**	**Cognitively preserved group** (*n* = 45)	**Total** (*n* = 90)	***P*-value**
History of status epilepticus	Yes	23 (51.1%)	14 (31.1%)	37 (41.1%)	0.087
	No	22 (48.9%)	31 (68.9%)	53 (58.9%)	
Neurological deficit	Yes	28 (62.2%)	23 (51.1%)	51 (56.7%)	0.395
	No	17 (37.8%)	22 (48.9%)	39 (43.3%)	
Epilepsy type	Occipital	3 (6.7%)	1 (2.2%)	4 (4.4%)	0.099
	Frontal	21 (46.7%)	32 (71.1%)	53 (58.9%)	
	Temporal	20 (44.4%)	12 (26.7%)	32 (35.6%)	
	Parietal	1 (2.2%)	0 (0.0%)	1 (1.1%)

**Table 3:** Interictal EEG findings by group

**EEG findings**	**Cognitively impaired group (***n* = 45**)**	**Cognitively preserved group (***n* = 45**)**	**Total (***n* = 90**)**	***P*-value**	Odds ratio^a^
Abnormal	35 (77.8%)	26 (57.8%)	61 (67.8%)	0.041	2.56 (1.02–6.41)
Normal	10 (22.2%)	19 (42.2%)	29 (32.2%)		

^a^OD Ratio is 95% Confidence Interval

### Multivariable predictors of cognitive impairment

**Fig. 1:**
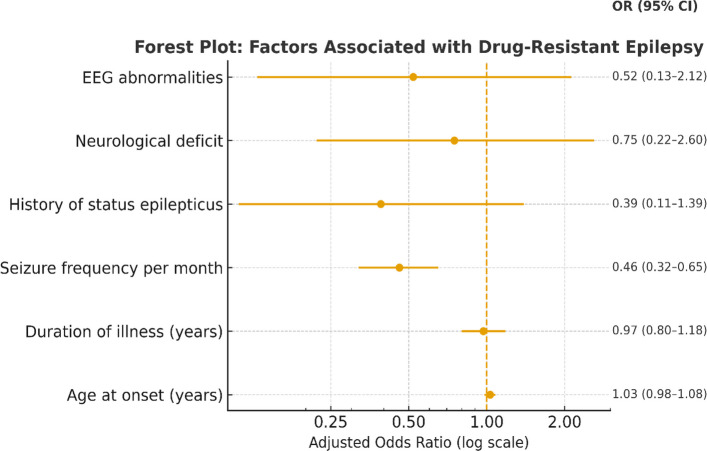
A forest plot describing factors associated with DRE

Seizure frequency per month emerged as the sole independent predictor of cognitive impairment. As cognitive preservation was used as the reference outcome in the regression model, odds ratios below unity indicate an increased likelihood of cognitive impairment. An additional monthly seizure would increase the likelihood of cognitive impairment by approximately 54% (adjusted OR = 0.46, 95% CI: 0.32–0.65, *P* < 0.001; values < 1 indicate inverse coding for preserved cognition). In contrast, EEG abnormalities, age at onset, illness duration, history of status epilepticus, neurological deficits, and epilepsy type did not show independent associations with cognitive impairment. Although interictal EEG abnormalities correlate with the cognitive status in univariate analyses, seizure burden remained the dominant explanatory factor after multivariable adjustment. The logistic regression results are summarized in Table 4 and Fig. 1.

**Table 4:** Multivariate logistic regression analysis of factors associated with DRE

**Variable**	**Adjusted OR** ^Exp(B)^	**95% CI for OR**	***P*-value**
Age at onset (years)	1.03	0.98–1.08	0.259
Duration of illness (years)	0.97	0.80–1.18	0.768
Seizure frequency per month	0.46	0.32–0.65	< 0.001
History of status epilepticus	0.39	0.11–1.39	0.145
Neurological deficit	0.75	0.22–2.60	0.647
EEG abnormalities	0.52	0.13–2.12	0.360

*Exp (B)* Exponential of Beta, *OD* Odd Ratio, *EEG* Electroencephalograph

## Discussion

This study examined neurophysiological and clinical correlates of CI in adults with DRE. The findings demonstrate that patients with CI exhibited significantly higher seizure frequency compared to cognitively preserved individuals. Although abnormal interictal EEG patterns were more prevalent in the impaired group, seizure burden remained the only independent predictor after adjustment for confounders. These results indicate that seizure frequency, rather than interictal EEG abnormalities, is the principal determinant of CI in DRE.

A higher proportion of CI patients reported a history of status epilepticus, although the difference was not statistically significant (Table 2). This observation remains clinically relevant, given that status epilepticus has been linked to hippocampal injury, neuronal loss, and maladaptive network reorganization contributing to both pharmacoresistance and long-term neurocognitive deficits [23, 24]. Similarly, neurological deficits occurred more frequently in cognitively impaired patients, aligning with evidence that structural abnormalities such as cortical dysplasia or hippocampal sclerosis are associated with poorer seizure control and unfavorable cognitive outcomes [25, 26]. While these variables did not independently predict cognitive status, their distribution supports seizure burden as a composite marker of disease severity.

Epilepsy type distribution also differed between groups (Table 2). Temporal lobe epilepsy was more common among cognitively impaired patients, whereas frontal lobe epilepsy predominated in those with preserved cognition. This pattern reinforces earlier evidence that temporal lobe epilepsy, particularly in refractory forms, is strongly associated with cognitive morbidity, especially affecting memory and learning domains [27, 28]. By contrast, frontal lobe epilepsy, while clinically disruptive, is generally associated with better treatment outcomes and relatively preserved cognition, further highlighting the clinical relevance of seizure localization.

Interictal EEG abnormalities were observed significantly more often in patients with CI, but this association was no longer significant after controlling for seizure frequency and other variables (Table 3). This suggests that routine interictal EEG findings primarily reflect underlying cortical dysfunction rather than independently influencing cognitive outcomes. Prior studies have yielded conflicting results, with some linking frequent interictal discharges to cognitive deficits, while others identified seizure burden as the dominant determinant [7, 10, 29]. In this context, our findings do not support EEG abnormalities as independent predictive markers; rather, they appear to be secondary indicators accompanying more severe epilepsy phenotypes.

Seizure frequency emerged as the strongest and only independent determinant of CI in this cohort. Patients with more frequent seizures exhibited significantly lower MoCA scores, consistent with previous evidence associating uncontrolled seizures with hippocampal sclerosis, network reorganization, and diffuse neurocognitive dysfunction [27, 30]. These results underscore that effective seizure control is crucial for maintaining cognitive function and emphasize the need for aggressive therapeutic optimization in refractory epilepsy. Furthermore the magnitude of CI observed across our cohort accentuates the burden of neuropsychiartric comorbidity in DRE. As reported elsewhere, deficits in attention, executive functioning, and memory appear particularly vulnerable to recurrent seizures [30, 31]. The robust association between seizure burden and MoCA scores in this cohort emphasizes the importance of incorporating routine cognitive screening into epilepsy management to facilitate early detection and timely rehabilitation interventions.

### Limitation

This study has several important limitations that should be considered when interpreting the findings. First, the cross-sectional design precludes causal inference, and longitudinal studies are required to determine whether interictal EEG abnormalities precede cognitive decline or merely reflect disease severity. Second, seizure frequency was based on clinical reporting, which may introduce recall bias. Third, EEG assessment relied on routine 20–30-min interictal recordings, potentially underestimating epileptiform activity compared with prolonged or sleep-deprived EEG monitoring. Fourth, standard clinical filter settings with a lower cutoff of 1 Hz were used; therefore, infra-slow EEG activity, which may carry additional pathophysiological relevance, was not evaluated. Fifth, cognitive function was assessed using the MoCA, a validated screening tool that does not provide domain-specific cognitive profiling. Sixth, quantitative EEG metrics were not extracted, limiting mechanistic interpretation. Finally, anti-seizure medication burden, including specific drug classes, dosing, and polytherapy effects, was not incorporated into the analysis. Despite these limitations, the study reinforces seizure burden as the dominant determinant of CI in DRE and supports the need for multimodal, longitudinal research.

### Future considerations and directions

Our study added to the clinical prediction of cognition in patient with DRE, but future work should refine both neurophysiological and cognitive assessments to better explain mechanisms underlying CI in DRE. A more granular characterization of interictal EEG abnormalities is warranted, including distinguishing epileptiform discharges from background slowing, quantificating discharge burden, and incorporating spatial or regional EEG features. Such approaches may clarify whether specific electrophysiological patterns convey information beyond general indicators of disease severity. Additionally, shifting from a binary definition to continuous cognitive measures of CI could yield more precise associations with EEG findings and seizure localization. Hence, longitudinal study designs are required to determine whether interictal EEG abnormalities have prognostic value for future cognitive decline rather than reflecting concurrent disease burden. Moreover, comprehensive adjustment for treatment-related variables, including anti-seizure medication will be essential to distinguish the relative contributions of seizures, treatment effects, and underlying network dysfunction to cognitive outcomes in DRE.

## Conclusions

In adults with drug-resistant epilepsy, seizure frequency was the primary determinant of cognitive impairment, underscoring the adverse effects of uncontrolled seizures on brain function and neurocognitive outcomes. Although interictal EEG abnormalities were more common in cognitively impaired patients, they did not independently predict cognition after adjusting for seizure burden and clinical factors. These findings indicate that EEG abnormalities should be interpreted alongside clinical indicators rather than in isolation. Clinically, achieving optimal seizure control remains central to preserving cognition, while EEG may complement risk assessment. Future longitudinal studies combining EEG, neuroimaging, and detailed neuropsychological testing are needed to refine prognostic models and guide early intervention.

## Data Availability

The datasets generated during and/or analysed during the current study are available from the corresponding author [MK] on reasonable request.

## Abbreviations

CI: Cognitive Impairment
DRE: Drug-Resistant Epilepsy
EEG: Electroencephalography / Electroencephalogram
MoCA: Montreal Cognitive Assessment
OR: Odds Ratio
CI (statistical): Confidence Interval
DNL: De Novo Lipogenesis
ILAE: International League Against Epilepsy
SSBs: Sugar-Sweetened Beverages
SPSS: Statistical Package for the Social Sciences
Hz: Hertz
mΩ: Milliohm
min: Minutes
